# Deprotonation Mechanism of Methyl Gallate: UV Spectroscopic and Computational Studies

**DOI:** 10.3390/ijms19103111

**Published:** 2018-10-11

**Authors:** Liangliang Zhang, Yuchen Liu, Yongmei Wang

**Affiliations:** Institute of Chemical Industry of Forest Products, Chinese Academy of Forestry, Nanjing 210042, China; hnsmxlyc@icifp.cn (Y.L.); njlhswym@icifp.cn (Y.W.)

**Keywords:** methyl gallate, polyphenol, tannins, pH titration, spectroscopic method

## Abstract

In the present paper, methyl gallate (MeG), a simple polyphenol and also the monomer of hydrolysable tannins, was selected to study the deprotonation process for the hydroxyls of the galloyl group by the combined use of spectroscopic measurements and quantum chemical calculations. The results of quantum chemical calculations show that the deprotonated form of methyl gallate undergoes the para-quinoid localization in the benzene ring, compared with free methyl gallate. The predicted spectra obtained from the free and deprotonated methyl gallate models are in agreement with the experimental UV-visible (UV-vis) absorption spectra. In the same way, the vibrational spectra of the para-quinoid MeG models validate the proposed mechanism of the deprotonation of MeG molecule. The pH influence on the deprotonation reaction and oxidization of phenolic groups has been also investigated. The p*K*_a_ values of MeG were evaluated using the chemometric modeling method. The first acid dissociation constant (p*K*_a1_) for MeG was evaluated to be 4.20 ± 0.01, and the second one (p*K*_a2_) was 10.78 ± 0.06.

## 1. Introduction

**Figure 1:**
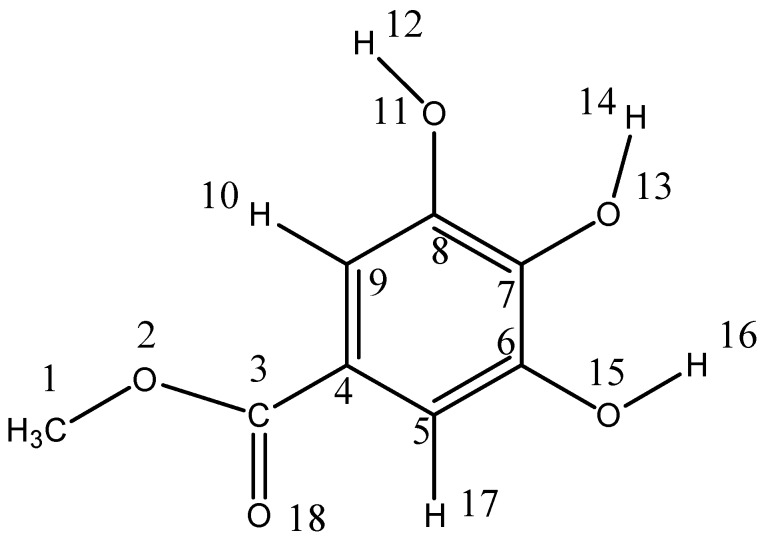
Structure and atomic numbering of methyl gallate.

The structure of plant tannins has a large number of phenolic groups that can be deprotonated and form complexes with metals, especially transition metals [1]. Tannin–metal binding interactions play an important role in regulating the nutrients and toxins in the soil for the plants and also affect various environmental processes such as formation of the humic substances [2]. Coordination of tannins with metal ions has been suggested through the phenolic groups based on the metal coordination properties of the simpler phenolics, such as methyl gallate and catechin [3,4,5]. A large number of studies [6,7,8,9] have shown that the chelation of metal ions by phenolic compounds preferentially occurs at the deprotonated catechol site. It is well known that the catechol moiety is a powerful complexing agent for some metal ions, especially for transition metal ions and, despite its extremely high acid dissociation constant (p*K*_a_) values, this function is able to coordinate metal ions at very low pH with a complete deprotonation of the hydroxyl groups. However, there has been no detailed study on deprotonation reaction of phenolic groups in the molecular structure of polyphenols and tannins. There are still many questions that need to be answered. For example, (I) whether the phenolic hydroxyls in the galloyl moiety deprotonated simultaneously? If not, which phenolic hydroxyl is much easier to be deprotonated during metal binding reactions? (II) Is there is an oxidization reaction happening during the deprotonation reaction? (III) Can the phenolic groups can be oxidized to be quinoid structures at the higher pH? In this paper, a simple phenolic compound methyl gallate, which could be regarded as the monomer of hydrolysable tannins, was used as a model compound to study the deprotonation mechanism of phenolic groups in methyl gallate. The representation of the methyl gallate molecule with the atomic numbering used in the text is given in Figure 1.

Identification or structural information of phenolic acids can be obtained by comparison of their UV-visible (UV-vis) absorption spectra in solution and in presence of metal or base, the observed spectral shifts of phenolic compounds in the solution are often characteristic of their substitution [8,10,11]. The metal-chelation mechanism by several phenolic acids has been studied by means of spectrophotometry and calculating the binding constants of the complexes [11]. The present study was conducted to study the deprotonation mechanism of phenolic groups in methyl gallate using the spectroscopic method. In order to better understand the structural changes of free and deprotonated methyl gallate molecules, quantum chemical calculations have been performed. Recent advances in computational chemistry techniques have made it possible to optimize the structures of small- and medium-sized compounds by density functional theory (DFT) calculations. Using DFT calculations, we were able to confirm and provide the modifications engendered by the deprotonation of phenolic groups in methyl gallate (MeG) molecule. The p*K*_a_ value of MeG is determined by pH titration method and the mechanism of deprotonation reaction of phenolic groups in MeG was proposed by fitting the computational data with experimental spectra.

## 2. Results and Discussion

### 2.1. pH Titration Study

**Figure 2:**
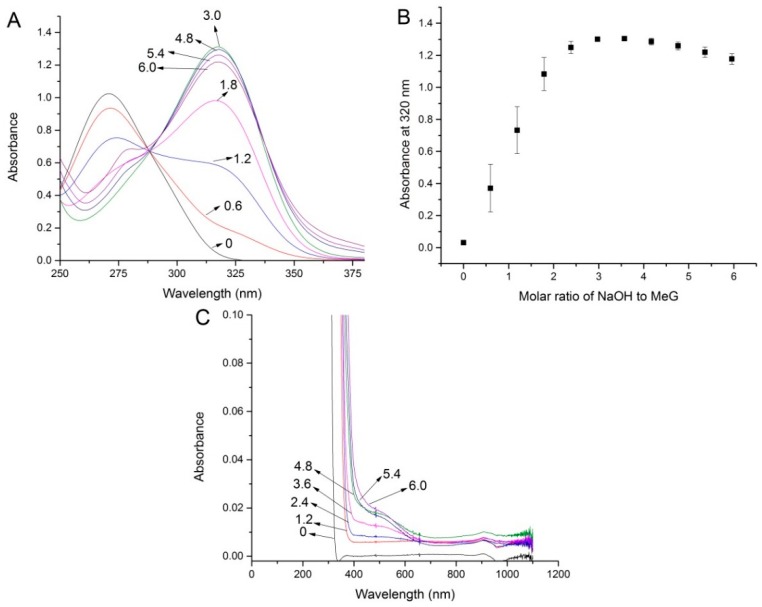
(**A**) UV-visible (UV-vis) spectra of methyl gallate solution continuously titrated with NaOH solution; (**B**) the absorbance changes at 320 nm, data are presented as mean ± SD; (**C**) the spectra changes at around 500 nm indicate a certain degree of oxidation of MeG; [NaOH]/[MeG] molar ratio are indicated on spectra.

Figure 2A shows a typical UV spectrum of pure MeG at pH 3.6 and the changes of the spectra when the solution was continuously titrated with NaOH solution. The spectrum of pH titration of MeG is showed in Figure 2A. As was observed by our previous study [12], pure MeG solution has a maximum absorbance at 275 nm. Following the NaOH addition, the spectra showed a distinct bathometic shift of the absorbance maximum towards 320 nm, indicating that there was a deprotonation reaction of the phenolic hydroxyl in MeG. The bathochromic shifts of the MeG spectrum are the result of the deprotonation reaction of phenolic hydroxyl in galloyl moiety. Figure 2B exhibits that the spectra kept red-shift in proportion with the NaOH addition until a molar ratio of NaOH/MeG reached close to three, suggesting all three phenolic hydroxyl in MeG can be deprotonated. A three-fold molar excess of NaOH is necessary to obtain a full deprotonated galloyl group. When continued addition of more NaOH into MeG solution occurs (the ratio of NaOH to MeG becomes greater than 4.8), a decrease of the absorbance at 320 nm was observed (Figure 2B), indicating the deprotonation species of MeG that generated by addition of NaOH decreased and other reactions may have happened. Furthermore, it should be noted that when we focused on the spectrum changes range from 400 to 600 nm, a slight absorbance at approximate 500 nm was also observed when the mixture contains high concentration of NaOH (the ratio of NaOH/MeG > 3.6) (Figure 2C), and this can be explained by a certain degree of oxidation reaction of phenolic hydroxyls in galloyl moiety. According to the previous study, at higher pH values, phenolic hydroxyl in galloyl groups can be oxidized to form a quinonoid structure [1,13,14]. It was reported that hydrolysable tannins are susceptible to oxidative reactions that transformed the structure especially at high pH (>9) and will give a UV spectral rise at 400–600 nm [14]. This can be proved by the observation of the decrease of the absorbance at 320 nm when more NaOH was added into MeG solution (Figure 2B).

### 2.2. Computational Study

Bond lengths and bond angles are reported in Tables S1 and S2, respectively, for free and deprotonated MeG molecules and the geometrical parameters obtained with DFT calculation are presented. For bond lengths, the difference can be noticed for C(3)–C(4), C(3)–O(18), and C(6)–O(15) bond lengths, and especially in the C(7)–O(13) bond length. C(7)–O(13) bond lengths were calculated by DFT for free molecule of 1.369 and deprotonated molecule of 1.237, indicating the structure and electron in C(7) was modified. The C(7)–O(13) and C(6)–O(15) bonds have identical lengths in the free molecular, and an increase of 0.132 Å for the C(7)–O(13) bond length can be noticed. The C(6)–C(7)–O(13) angle value is enhanced by approximately 2.5°, and the C(7)–C(6)–O(15) is reduced by about 5.6° in the deprotonated form. All these changes mentioned above can be easily associated with the hydroxyl groups deprotonation mechanism induced by pH rise that removes the hydrogen bonding present in the free MeG molecule [9].

**Figure 3:**
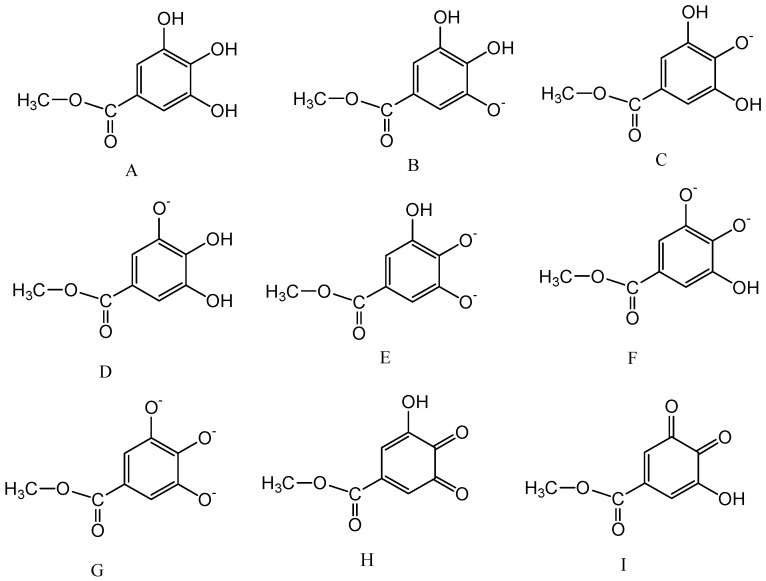
Proposed deprotonated and quinoid methyl gallate structures used to calculate spectra by Gaussian.

**Figure 4:**
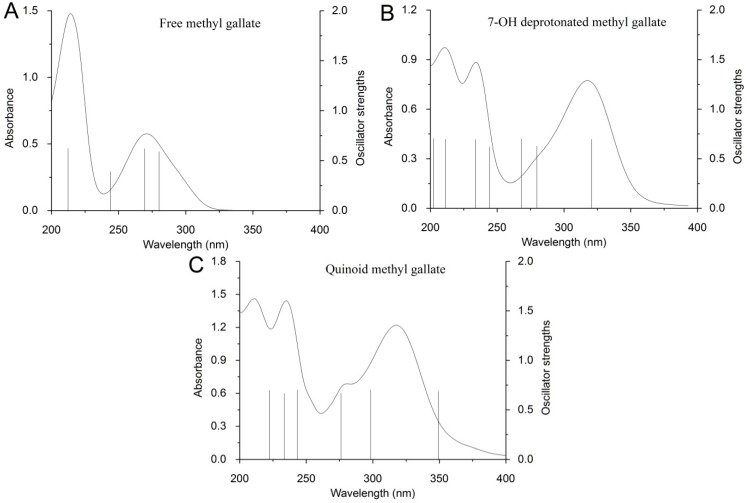
Computed transitions (vertical line) and experimental spectrum (curve) for (**A**) free methyl gallate, (**B**) 7-OH deprotonated methyl gallate, and (**C**) quinoid methyl gallate.

A computational method was also employed to figure out whether quinoid MeG can be formed when MeG solution contains high concentration of NaOH. Proposed deprotonated and quinoid MeG structures used to calculate spectra by Gaussian software are shown in Figure 3. The lowest energy transition wavelength and oscillator strength of the different protonation states of MeG and quinoid MeG are calculated by Gaussian software using time-dependent density functional theory level (TD-DFT) method. The calculated results are shown in Table 1. It can be seen that fully protonated MeG (free MeG) has a maximum absorbance of 279.84 nm, which is very close to the experimental wavelength of 275 nm. Furthermore, 7-OH deprotonated MeG (the structure is showed in Figure 3C) has a maximum absorbance of 320.25 nm, which is much closer to that of our experimental data (320 nm). Theoretical and experimental spectra of free and deprotonated MeG species are represented in Figure 4. The vertical line heights of theoretical spectra are relative to the values of oscillator strengths. The results from the present study indicated that in spite of solvent effects that must affect the absorption spectra, the theoretical absorption bands positions are relatively in good agreement with the experimentally observed ones. The previous study [9] suggested that the experimentally observed intensities of bands do not always have to be correctly reproduced by the calculations, demonstrating that theoretical calculations of electronic oscillator strengths have to be used very carefully. In the present study, the calculation results fairly reproduce the bathochromic shift of band I, which were experimentally observed in the spectra of deprotonated species.

**Figure 5:**
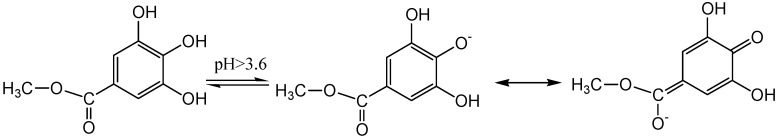
The para-quinoid resonance structure of the deprotonated methyl gallate system.

Figure 4 shows a good fitting of the computed data with experimental spectra for free and deprotonated methyl gallate. Based on the calculation data, 7-OH deprotonated MeG can be confirmed as the main deprotonated form and has been formed in the solution containing a ratio of NaOH to MeG of 2.4. The (-) charge of O(13) in structure C (Figure 3) can be delocalized to the O(18) (of C=O) through the benzenic ring, and this enhances the para-quinoid localization in the benzene ring. The corresponding electron (de)localization changes are also supported by most of the bond length changes presented in Tables S1 and S2. In the supplementary data, the bond lengths presented with bold characters show that at the deprotonated system, the C(3)–C(4) and C(7)–O(13) lengths diminish, while the C(3)–O(18) length increases. This is in agreement with the para-quinoid resonance structure of the deprotonated system exhibiting O(13)=C(7) and C(4)=C(3)–O(18)(-), which are responsible for the electron delocalization from O(13)–O(18). Moreover, the increasing of the lengths (from free to deprotonated system) of C(4)–C(5), C(4)–C(9), C(7)–C(6), and C(7)–C(8) corroborate the enhancement of the para-quinoid resonance structure. Based on this, the para-quinoid resonance structure of the deprotonated system and the changes in electronic delocalization are depicted in Figure 5. The spectra of different quinoid MeG were also calculated and 7,8-OH double quinoid MeG shows a good fitting with the experimental spectra of MeG solution, which contain a higher concentration of NaOH (ratio of NaOH to MeG of 6) (Figure 4C). Based on this, it can be concluded that the main deprotonation species is 7-OH deprotonated MeG and MeG can be oxidized to form 7,8-OH double quinoid MeG when the solution contains higher concentration of NaOH. Thus, it is reasonable to conclude that compared with other phenolic hydroxyl in MeG, 7,8-OH is more deprotonated in the high pHs.

Indeed, the quinoidal form of phenolic groups can be found in their complexation with metal ions. Brouillard and coworkers [15,16] have studied anthocyanins-Al (III) complexes, which involves a catechol moiety in the molecular structure. They have shown that the aluminium complexes of 3′,4′,7-trihydroxy-3-methoxy-flavylium chloride adopt a quinoidal form that is induced by the deprotonation of the two hydroxyl groups when Al (III) is coordinated to the catechol group. Cornard et al. [9] also confirmed that the hydroxyl function in position 4 of catechol group is conjugated to the 4-carbonyl group and leads, by deprotonation, to a quinoidal form.

### 2.3. Calculation of pK_a_ Values

**Figure 6:**
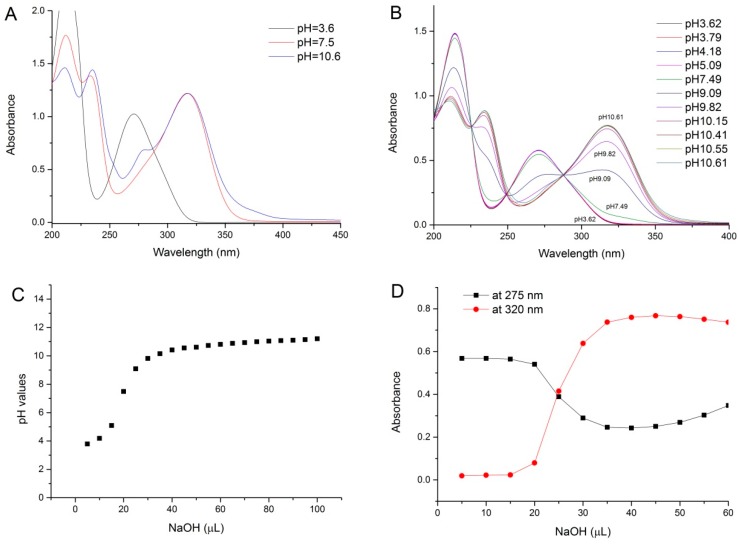
(**A**) pH of free and deprotonated methyl gallate; (**B**) evolution with pH of the UV-vis spectra of methyl gallate; (**C**) pH changes of methyl gallate solution with the increment of NaOH; (**D**) absorbance changes of methyl gallate solution at 320 nm and 275 nm with the increment of NaOH.

The pH values after each titration with NaOH are measured, and it was found that the pH value of free MeG is 3.6. When the 2.4-fold of NaOH was added into MeG, the pH increases to 7.5, and the pH reaches 10.6 when 6-fold of NaOH was added (Figure 6A). Thus, the deprotonation of MeG takes place from the pH of 3.6 to 7.5 and the oxidization happens from the pH of 7.5 to 10.6. In the case of pH values below 7.5, no oxidization was observed and the predominant specie is 7-OH deprotonated MeG. The evolution with pH of the UV-vis spectra of MeG titrated with NaOH is showed in Figure 6B. It can be seen from the figure that increasingly more MeG deprotonated with the increasing of pH. However, higher pH (10.6) can cause an oxidization of phenolic hydroxyl to form quinoid MeG. The pH changes of MeG solution with the increment of NaOH are shown in Figure 6C. There is a rapid increase of pH of MeG solution when 5–50 µL NaOH (the ratio of NaOH to MeG is 0.6–6) was added into the solution. After that, more NaOH does not cause an obvious change of pH values, indicating that almost all of the phenolic hydroxyls in methyl gallate are deprotonated. The p*K*_a_ values of methyl gallate can be determined by plotting absorbance changes of MeG solution at 320 and 275 nm with the increment of NaOH (Figure 6D). The pH at the intersection point of absorbance changes at 320 and 275 nm was determined to be 9.11, which can be considered as the p*K*_a_ value of methyl gallate.

### 2.4. Chemometric Modeling Study

**Figure 7:**
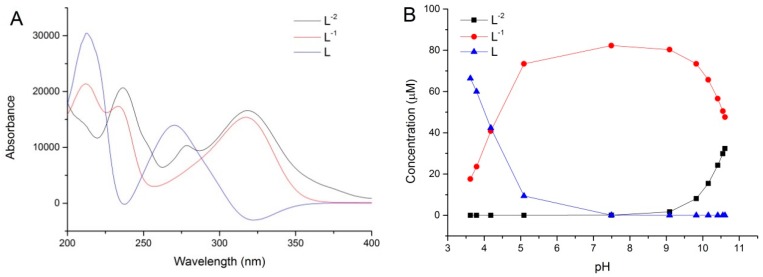
(**A**) The characteristic spectra of MeG and its two deprotonated species (L^−1^, L^−2^) predicted using chemometric modeling method; (**B**) concentration profiles of protonated MeG (L) and its two deprotonated species predicted by the chemometric method.

Based on the spectral changes of pH titration and the results of calculation mentioned above, it was confirmed that there are at least two deprotonation processes for methyl gallate observed during the titration process. The p*K*_a_ values of MeG were evaluated using chemometric modeling of the potentiometric and spectrophotometric titration data. The first acid dissociation constant (p*K*_a1_) for MeG was determined to be 4.20 ± 0.01, and the second one (p*K*_a2_) was 10.78 ± 0.06, which is similar to that obtained using potentiometric and spectrophotometric titration data (9.11). It should be noted that each p*K*_a_ corresponded to a unique spectral species, as shown in Figure 7A. The characteristic spectra of protonated MeG species (L) and its two deprotonated species (L^−1^, L^−2^) formed during the titration can be predicted using the chemometric modeling method (Figure 7A). The results showed that the predicted spectra fit the experimental data quite well (Figure S1). It can be seen from the Figure 7A that the absorption bands of protonated MeG species (L) are observed at 210 and 275 nm. The absorption bands of singly deprotonated species of MeG (L^−1^) are observed at 210, 230, and 320 nm. Compared with protonated MeG, both bands at 210 and 275 nm were weakened and new peaks appeared at 230 and 320 nm with an increase in pH. The double deprotonated species (L^−2^) had similar spectra changes to singly deprotonated species with an additional small peak appearing at 280 nm, which indicated an oxidization of phenolic hydroxyl of MeG. The concentration profiles of free and deprotonated MeG species in the mixtures have also been predicted by chemometric modeling and are shown in Figure 7B. It can be seen from the figure that the first deprotonation of MeG takes place from the pH of 3.6 to 7.5 and the second deprotonated one happens from the of pH 7.5 to 10.6. These results are in excellent agreement with the data obtained by the titration experiment in the present study. However, it should be noted that there are still approximately 47% of singly deprotonated species of MeG in the mixture in the end of titration. At this point, the content of double deprotonated species in the mixture is only around 32%. Based on this, the predominant species in the solution in the end of titration is singly deprotonated species of MeG.

## 3. Materials and Methods

### 3.1. Chemicals

Methyl gallate (MeG) was purchased from Sigma-Aldrich (St. Louis, MO, USA). The working solution of MeG (1.68 mM) was freshly prepared in 50% methanol–water solution before each titration, and the exact concentration was determined using the extinction coefficients at 274 nm (11.8 M^−1^ cm^−1^). The working solution of NaOH (10 mM) was prepared in water. Metal-free water was used throughout the experiments and all other chemicals were of reagent grade or better.

### 3.2. Instrumentation

The UV-vis spectra were collected for free MeG and its deprotonated species using an Agilent 8454 UV-vis spectrophotometer (Agilent, Santa Clara, CA, USA) and analysed using ChemStation Software (Revision B.05.03, Agilent, Santa Clara, CA, USA). The pH of the solutions was measured with a Mettler Toledo pH meter (model S210) using a combined electrode (Mettler Toledo, Shanghai, China).

### 3.3. pH Titrations

pH titrations were performed manually with a pipette. Fifty microliters of 1.68 mM MeG was mixed with 950 μL deionized water in a 1 mL quartz cuvette with a pathlength and were titrated with 10 mM NaOH solution in increments of 5 μL at room temperature (22 °C). To determine the p*K*_a_ value of MeG, the stock solution of MeG was diluted to 84 μM with small amount of diluted hydrochloric acid solution in cuvette and titrated with NaOH solution as described above. UV-vis spectra from 190–600 nm were recorded after each addition. The pH was recorded when a stable reading was reached, after 1–3 min after each step of the titration, and was carried out until the pH of the mixture reached near 12. The experiments were repeated three times, and the potentiometric and spectrophotometric titration data could be used to calculate the p*K*_a_ value of MeG. The titration data were also analysed by chemometric methods using Reactlab Equilibrium software (JPlus Consulting http://jplusconsulting.com/products/reactlab-equilibria/), and the p*K*_a_ values of MeG were evaluated.

### 3.4. Computational Method

All computations were performed using Gaussian 09 program (Revision A.02, Gaussian, Inc., Wallingford, CT, USA). Bond lengths, bond angles, excitation energies, and oscillator strengths for the protonated and deprotonated MeG molecules were computed at the time-dependent density functional theory (TD-DFT) level, using the B3LYP functional. Full geometry optimization was carried out using the Gaussian 09 program package, employing density functional theory method B3LYP [17,18] with the 6-31G(d) basis set [19]. Solvent effects were modeled with water as the solvent using the polarizable continuum model (PCM) [20]. The TD-DFT method has gained increasing popularity among chemistry scientists because of its ability to reproduce absorption spectra of both small compounds and its complexes [9,12,13,14,15,16,17,18,19,20,21].

## 4. Conclusions

The structural changes caused by deprotonation reaction of the two hydroxyls of the galloyl group were studied in the present paper. Based on the computational data, 7-OH deprotonated MeG is formed with the increment of NaOH and 7,8-OH double quinoid MeG is formed under the condition of more NaOH being added into the solution. This study illustrates that with the increase of pH of MeG solution, 7-OH deprotonated MeG is formed, but higher pH (10.6) can cause an oxidization of phenolic hydroxyl to form 7,8-OH double quinoid MeG. The p*K*_a_ values of MeG were determined, using chemometric modeling, to be 4.20 ± 0.01 and 10.78 ± 0.06, respectively, for the first acid dissociation constant (p*K*_a1_) as well as the second one (p*K*_a2_). This study can help researchers to better understand the binding reaction of phenolic compounds with metal ions. However, how this deprotonation of phenolic compounds affects their binding capacity with metal ions still needs to investigated in the future.

## Figures and Tables

**Table 1:** Lowest energy transition wavelength and oscillator strength calculated for the different protonation states of methyl gallate and quinoid methyl gallate.

Considered Form	Wavelength (nm)	Transition Energy (eV)	Oscillator Strength
MeG fully protonated, A	279.84	4.4305	0.59652
MeG 6-OH deprotonated, B	392.35	3.16	0.70062
MeG 7-OH deprotonated, C	320.25	3.8715	0.69726
MeG 8-OH deprotonated, D	366.87	3.3795	0.6963
MeG 6,7-OH deprotonated, E	429.8	2.8847	0.68639
MeG 7,8-OH deprotonated, F	419.25	2.9573	0.68916
MeG fully deprotonated, G	430.4	2.8807	0.707
Oxidate MeG, H	429.76	2.8849	0.68639
Oxidate MeG, I	423.56	2.9272	0.68758

Notes: Structures are indicated in Figure 3. All the structures were calculated using the exact same parameters with the time-dependent density functional theory level (TD-DFT) method and solvent water. MeG—methyl gallate.

## Supplementary Materials

Supplementary materials can be found at http://www.mdpi.com/1422-0067/19/10/3111/s1.

## Abbreviations

MeG	Methyl gallate
TD-DFT	Time-dependent density functional theory
p*K*_a_	Acid dissociation constant
