# Supplementary material for: Deprotonation Mechanism of Methyl Gallate: UV Spectroscopic and Computational Studies
Source: Int J Mol Sci. 2018 Oct 11;19(10):3111. doi: 10.3390/ijms19103111 (PMC6213618; doi:10.3390/ijms19103111)
Supplement: Supplementary file 1 [file ijms-19-03111-s001.docx]

Supplemental Table 1 Bond length (Å) calculated by the DFT method for free and deprotonated methyl gallate

|  | Free molecule | Deprotonated molecule |
| --- | --- | --- |
| C(1)O(2) | 1.434 | 1.415 |
| O(2)C(3) | 1.358 | 1.364 |
| **C(3)C(4)** | **1.486** | **1.381** |
| **C(3)O(18)** | **1.216** | **1.357** |
| C(4)C(5) | 1.400 | 1.444 |
| C(5)C(6) | 1.392 | 1.361 |
| C(6)C(7) | 1.400 | 1.483 |
| **C(6)O(15)** | **1.362** | **1.385** |
| C(7)C(8) | 1.398 | 1.534 |
| **C(7)O(13)** | **1.369** | **1.237** |
| C(8)C(9) | 1.389 | 1.411 |
| C(8)O(11) | 1.378 | 1.258 |
| C(9)C(4) | 1.404 | 1.441 |
| O(13)H(14) | 0.972 |  |
| O(15)H(16) | 0.973 | 1.441 |

Supplemental Table 2 Bond angles (°) calculated by the DFT method for free and deprotonated methyl gallate

|  | Free molecule | Deprotonated molecule |
| --- | --- | --- |
| C(1)O(2)C(3) | 115.028 | 114.580 |
| **O(2)C(3)C(4)** | **112.464** | **124.477** |
| **O(2)C(3)O(18)** | **122.702** | **112.191** |
| C(4)C(3)O(18) | 124.834 | 123.331 |
| C(3)C(4)C(5) | 117.538 | 118.489 |
| C(3)C(4)C(9) | 121.752 | 122.449 |
| C(5)C(4)C(9) | 120.710 | 118.994 |
| C(4)C(5)C(6) | 119.831 | 120.487 |
| C(4)C(5)H(17) | 120.052 | 118.510 |
| C(6)C(5)H(17) | 120.117 | 120.979 |
| C(5)C(6)C(7) | 119.660 | 122.741 |
| C(5)C(6)O(15) | 120.313 | 122.792 |
| **C(7)C(6)O(15)** | **120.027** | **114.462** |
| C(6)C(7)C(8) | 120.225 | 117.625 |
| **C(6)C(7)O(13)** | **117.649** | **120.061** |
| C(8)C(7)O(13) | 122.127 | 122.313 |
| C(7)C(8)C(9) | 120.558 | 115.715 |
| C(7)C(8)O(11) | 114.490 | 117.980 |
| C(9)C(8)O(11) | 124.952 | 126.298 |
| C(4)C(9)C(8) | 119.016 | 124.342 |
| C(8)O(11)H(12) | 109.492 |  |
| C(7)O(13)H(14) | 107.877 |  |
| C(6)O(15)H(16) | 107.716 | 106.472 |


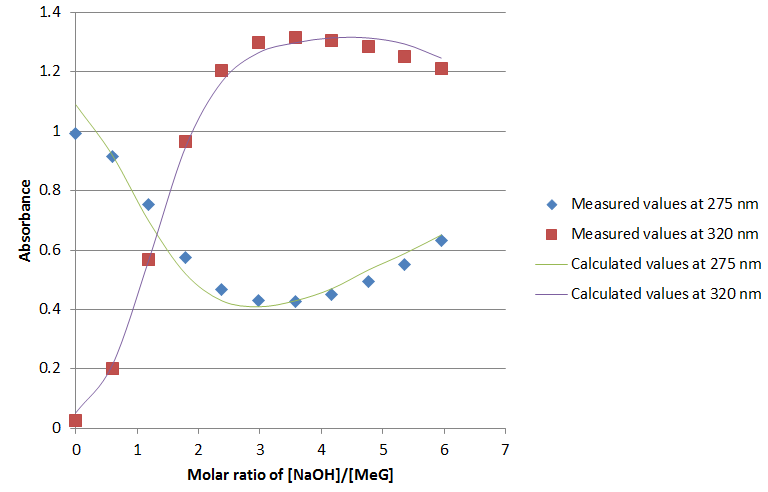


Supplemental Figure 1 Fitting plots of chemometric modeling of spectrophotometric titration data.
